# Calculation method to predict postoperative limb length in patients undergoing THA following developmental dysplasia of hips

**DOI:** 10.1186/s12891-019-2903-7

**Published:** 2019-11-03

**Authors:** Renwen Guo, Jerry Yongqiang Chen, Guoqiang Zhang, Yonggang Zhou, Jiying Chen, Wei Chai

**Affiliations:** 1 Department of Orthopaedic Surgery, Chinese People’s Liberation Army General Hospital, 28 Fuxing Road, Beijing, 100853 People’s Republic of China; 2 Department of Orthopaedic Surgery, Singapore General Hospital, Outram Road, Singapore, 169608 Singapore

**Keywords:** Limb length discrepancy, Total hip arthroplasty, Dysplasia of the hip, Calculation method

## Abstract

**Background:**

Limb length discrepancy (LLD) is one of the main cause of dissatisfaction after total hip arthroplasty (THA). The teardrop-lesser trochanter method can accurately predict and analyze LLD for healthy people. However, for patients with preoperative LLD, no method for predicting postoperative LLD is currently available, and these patients are highly susceptible to more severe LLD after THA. Accordingly, this study proposed a calculation method to predict postoperative limb length for these patients.

**Methods:**

Eighty patients who underwent THA between May 2016 and October 2018 due to unilateral developmental dysplasia of the hip (DDH) were evaluated. Relevant parameters were measured from radiographs of full-length lower limbs, e.g. the distance between the rotation center of the hip and the midpoint of the tibial plafond and the distance between the point which was marked at the same height as the lesser trochanter on the anatomical long axis of the femur and the midpoint of the tibial plafond. Then, a mathematical model was established by simplifying the structure from the hip to the ankle. The relationship between the placement position of the prosthesis and the LLD value was calculated by Law of Sines and Iterative Calculation.

**Results:**

The preoperatively predicted LLD values and the postoperatively measured LLD values were compared, yielding a mean absolute difference of 3.7 (range, 0.1 to 8.6) mm. The intraclass correlation coefficient (ICC) of the two parameters exhibited strong reliability (*ICC* = 0.911, 95%CI, 0.795 to 0.955). The Bland-Altman plot also showed good conformity between the two parameters.

**Conclusions:**

The proposed calculation method effectively predicted the postoperative LLD using preoperative parameters. Despite the complexity of the method, it can go a long way towards reducing the occurrence of severe postoperative LLD in DDH-THA.

## Background

Total hip arthroplasty (THA) is a very successful joint replacement surgery that can effectively improve function and reduce the pain of patients [1–3]. However, THA may lead to some problems. For example, limb length discrepancy (LLD) is a common cause of dissatisfaction and lawsuits after THA [4–6]. Overt LLD can cause dysfunction and lower back pain, inducing significant discomfort for patients [7–16].

In healthy people, postoperative LLD occurs with THA when the prosthesis model and placement position are incompatible with the normal structure. For example, a simple too long shaft component often leads to LLD. Accordingly, Ranawat et al. [17] proposed the teardrop-lesser trochanter method for evaluating limb length by radiographically measuring the perpendicular distance between the teardrops and the center point of the lesser trochanters. The teardrop-lesser trochanter method is widely used in surgical planning, as limb length can be predicted by template measurements. Also, this method has derived a variety of other methods for measuring the positional relationship between the pelvis and the femur. Computer-navigated surgery based on these methods can generally achieve an LLD value within 5 mm [18].

However, the teardrop-lesser trochanter method has a limited scope of application. The prerequisite of the method is that no accompanying gross deformity, fixed pelvic tilt, or shortening of the affected limb at a site other than the hip are present [17]. In clinical practice, this method is not applicable to preoperative LLD caused by many diseases, including developmental dysplasia of the hip (DDH).

A new calculation method was designed, which can be used for making a plan to correct preoperative unequal lengths of lower limbs. It can calculate the position of the prosthesis to be implanted according to the target postoperative limb length. We compared the preoperatively predicted LLD with the postoperatively measured LLD in order to find out whether the new calculation method (with measurement method) was sufficiently accurate.

## Method

### Materials

The medical and radiographic data of our hospital were retrospectively reviewed between May 2016 and October 2018. The inclusion criteria were as follows: (1) patients who were diagnosed with unilateral osteoarthritis secondary to DDH and underwent unilateral THA, (2) cases involving modular S-ROM femoral prostheses (DePuy, Warsaw, IN). The exclusion criteria were as follows: (1) patients aged less than 18 years old, (2) cases with incomplete or low quality medical records, including radiographs of the full-length lower limbs preoperative, and postoperative (at least 3 months later), (3) patients with other diseases that could affect the hip, knee, and surrounding structures and stability, such as rheumatic diseases and severe knee osteoarthritis.

Eighty patients were included in this study, with a median age of 42.3 (range, 22 to 67) years, including 11 men and 69 women. Among them, 56 patients underwent a left THA while 24 underwent a right THA. The Crowe classes [19] I: II: III:IV were 22:15:7:36. Overall, 22 patients required a subtrochanteric transverse osteotomy.

### Surgical information

The femoral prosthesis used for all patients was S-ROM prostheses, and the acetabular prostheses were PINNACLE acetabular cups (DePuy) or the CombiCup acetabular systems (LINK, Hamburg, Germany) with an appropriate diameter. The interfaces of the prostheses were ceramic on ceramic.

All operations were performed via the posterolateral approach and standard THA procedure [20]. Acetabular prosthesis was installed before femoral prosthesis. LLD was estimated by comparing the positions of knee joints and ankle joints after trials insertion and reduction of the hip. After adequate soft tissue and tendon release, if reduction of the hip still cannot be achieved, then a subtrochanteric transverse osteotomy was performed [20, 21].

### Radiographic measurement

**Fig. 1:**
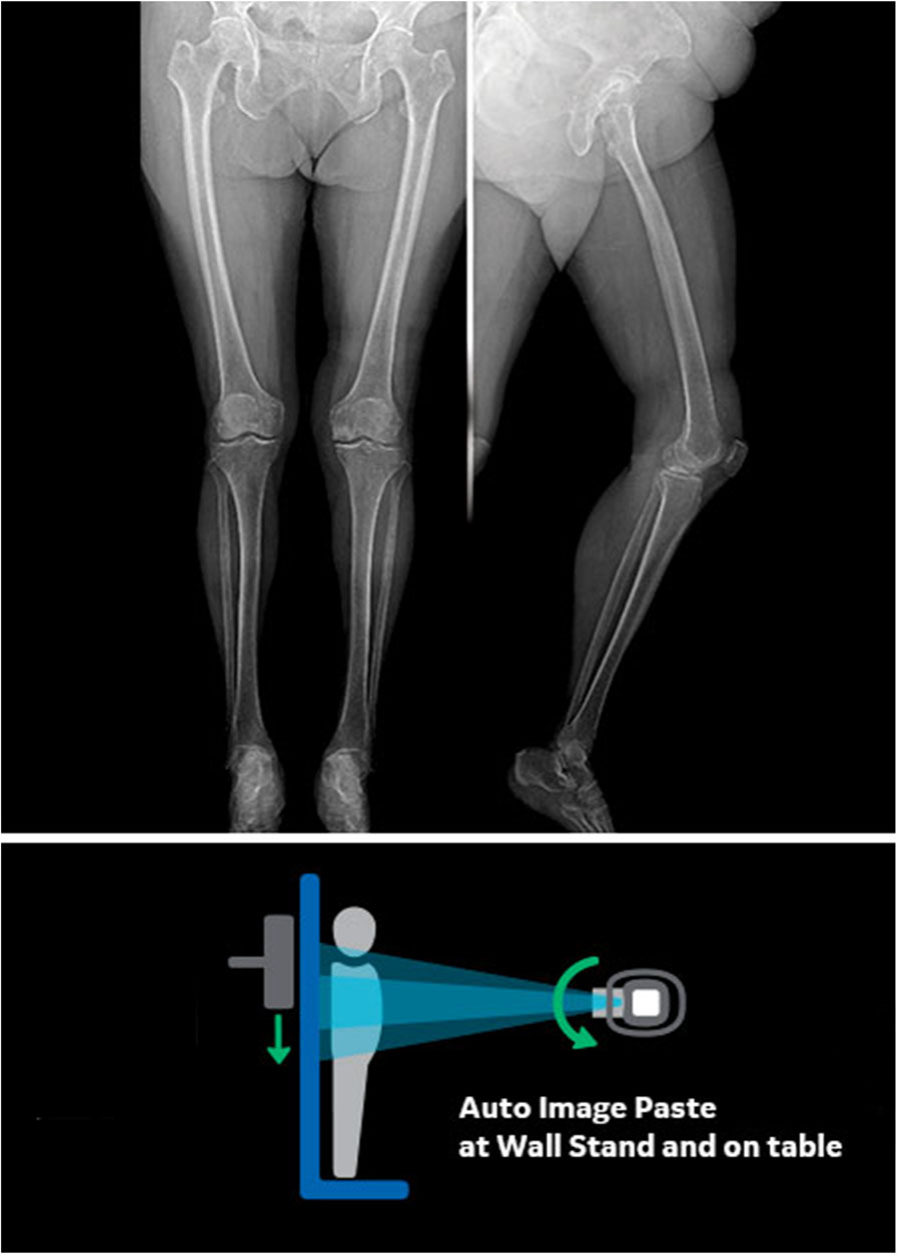
XR656 automatic image splicing to obtain a full-length image (authorized and reproduced from the digital image system introduction website)

Revolution XR656 digital imaging system (GE Healthcare) was used in this study to capture relevant images with a standard process [22, 23], including radiographs of the full-length lower limbs preoperative and postoperative (at least 3 months later). The digital imaging system had built-in full-length film shooting mode and Auto Image Paste (AIP) program (Fig. 1).

**Fig. 2:**
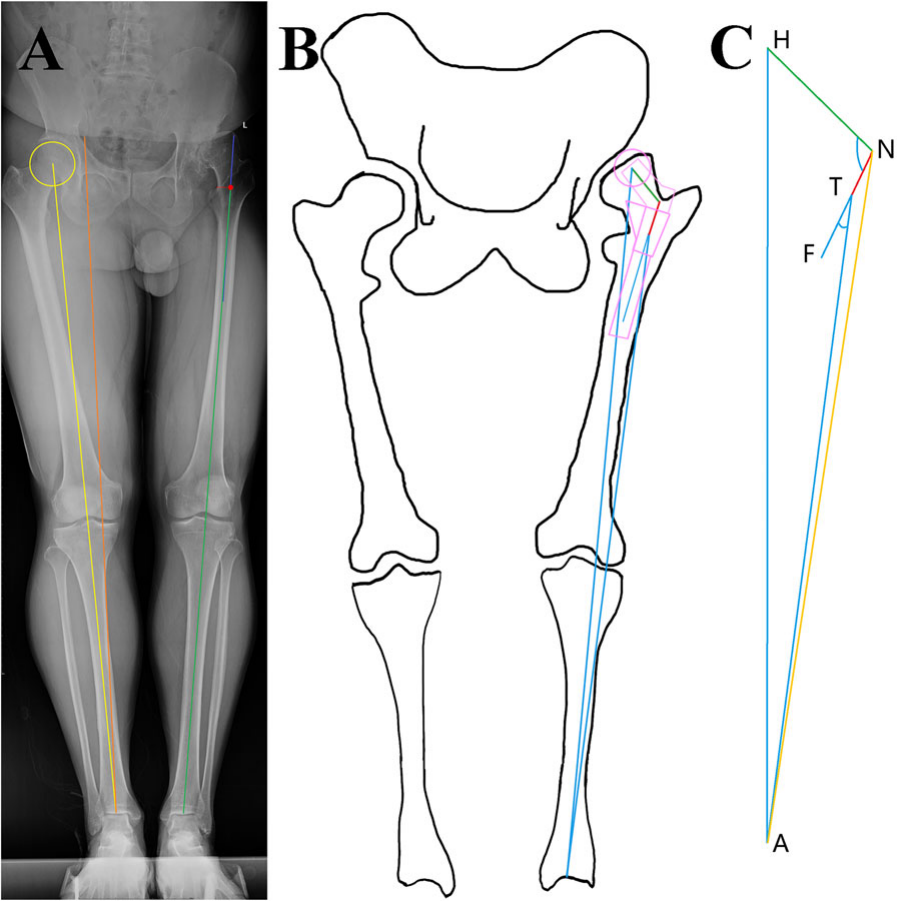
Schematic diagram of the calcuation method. **a** The blue line is the proximal axis of the femur on the affected side, the red dot is the projection point T of the upper edge of the lesser trochanter on the proximal midpoint of the femur, the green line is the TA, the orange line is the RLL, the yellow line is the LL. **b** and **c** The blue color designates the known line segment and angle, green designates the neck length of the appropriate prosthesis models, and the red segment NT is the target to be solved

The radiographs were viewed and measured on a picture archiving and communication system (PACS, UniWeb Viewer, version 7.0; EBM Technologies, China). The acetabular prosthesis was used to calibrate the magnification in the postoperative image, and the measured diameter of the best-fit circle aligned with the contralateral femoral head margin in the preoperative image was used for correction of magnification. Some parameters were defined, calibrated, and measured (Fig. 2a).

1. The center of the femoral head serves as the hip rotation center *H* (hip center).
2. The anatomical proximal axis of the femur is drawn. A point 100 mm below the most prominent part of lesser trochanter on the anatomical proximal axis of the femur is defined as *F*. The neck and stem axes (also the femur axis) intersection of the planed S-ROM prosthesis is defined as *N* (neck). The position of *N* can be calculated later.
3. Select a bony landmark in the intertrochanteric region that can be accurately located in X-ray and surgery. The landmark can be the most medial prominence of the lesser trochanter, or the upper edge of the lesser trochanter, or the top of the greater trochanter. Point *T* (trochanter) refers to a point on the anatomical proximal axis of the femur and at the same height as the landmark.
4. The midpoint of the tibial plafond is *A* (ankle).
5. The limb length (LL) is the distance between the center of hip rotation and the midpoint of the tibial plafond (*HA*).
6. The relative limb length (RLL) is the distance between the lower edge of the sacroiliac joint and the midpoint of the tibial plafond [24]. The difference between the affected and contralateral RLL is the RLL discrepancy (RLLD).

Two well-trained and experienced surgeons (G.R. and Y.M.) performed separate measurements, and the results were averaged. When the RLLD values they measured were compared, the interobserver reliability of the radiographic measurements indicated strong agreement, and the intraclass correlation coefficient of the RLLD was 0.987.

### Limb length calculation method

This method calculated the relationship between the specific position of the prosthesis and the length of the lower limb through sine theorem in two triangles. The height of the prosthesis installed (*NT*) was obtained by the following steps, as shown in Fig. 2b and c. The S-ROM prosthesis was used as an example.

1. Template measurement was conducted to determine the position of the acetabular prosthesis. The target limb length was calculated and the height difference between centers of rotation (COR) of the joints on both sides was measured. The higher the COR position, the longer the target leg.
2. Δ*HNA* and Δ*NTA* were drawn, and ∠*TAN* to *x* was set. ∠*ATF*(*a*), ∠*HNT* (the actual neck angle of the S-ROM prosthesis is 135°), *HA*, and *TA* were already known.
3. As ∠*HNA*, *HN*, and *HA* in Δ*HNA* were known, *NA* could be obtained from the sine theorem.
4. With ∠*NTA* (180° - *a*), *NA*, and *TA* in Δ*NTA* known, ∠*ANT* could be obtained from the sine theorem. The following equation could be derived.

$$ {x}_{n+1}=\alpha {\hbox{-} \sin}^{\hbox{-} 1}\left\langle \frac{TA\times \sin \alpha \times \sin \left(45{}^{\circ}-\alpha +{x}_n\right)}{HA\times \sin \left\{45{}^{\circ}-\alpha +{x}_n-{\sin}^{-1}\left[ HN/ HA\times \sin \left(45{}^{\circ}-\alpha +{x}_n\right)\right]\right\}}\right\rangle $$

5. The initial value of *x* was set to 1°, and the angle value with the required precision could be obtained after 2 iterations of calculation.
6. The length of *NT* in Δ*NTA* was determined with known ∠*TAN*, ∠*TNA*, and *TA* in Δ*NTA*. According to the prosthesis information plus the appropriate height, the distance between the prosthesis shoulder and the selected bony landmark could be obtained.

Based on the above method, this study reverse-calculated the length of the lower limb on the affected side based on relevant imaging data and recorded prosthetic parameters and prosthetic position parameters (including osteotomy length if it had been performed). An excel file in Additional file can help related calculation.

### Data processing and statistical methods

The medical records of these patients and follow-up data were reviewed. The prosthesis parameters of the patients were recorded, and the limb length and relevant parameters before and after surgery were measured. After measurement and calculation, the following parameters were obtained:

1. LLD_predicted_: on preoperative images, the limb length parameters, prosthesis information, and intended placement position were used to predict the postoperative length of the affected limb, and then the length of the contralateral limb was subtracted to obtain LLD_predicted_.
2. LLD_calculated_: on postoperative images, the limb length parameters, prosthesis information, and placement position were used to calculate the postoperative length of the affected limb, and then the length of the contralateral limb was subtracted to obtain LLD_calculated_.
3. LLD_true_: on postoperative images, the actual postoperative limb length difference LLD_true_ was measured and calculated.
4. The error of the calculate method itself: the difference between the LLD_calculated_ and LLD_true_.
5. The total error: the difference between the LLD_predicted_ and LLD_true_.
6. RLLD: in addition, the way operations reduced the True Limb Length Discrepancy (True LLD) was evaluated by the change in RLLD values.

The data from this study was statistically analyzed using MedCalc Statistical Software version 18.2.1 (MedCalc Software bvba, Ostend, Belgium; http://www.medcalc.org; 2018). The reliability test was performed by drawing the Bland-Altman plot [25], and calculating the Intraclass Correlation Coefficient (ICC). The ICC < 0.4 was considered poor reliability, 0.4 ≤ ICC < 0.6 was fair reliability, 0.6 ≤ ICC < 0.75 was good reliability, and ICC ≥ 0.75 was excellent reliability. Paired Student *t*-test was performed on the RLLD values. The statistical test level was α = 0.05. Clinically, an LLD value ≤10 mm was considered insignificant [14, 16].

## Results

### Reliability of LLDs

The mean value was 2.8 (SD, 10.6) mm for LLD_predicted_, 1.2 (SD, 11.7) mm for LLD_calculated_, and 2.4 (SD, 10.7) mm for LLD_true_. The ICC (evaluated with 2-way random effects model) of LLD_calculated_ and LLD_true_ was calculated to be 0.960 (95%CI, 0.928 to 0.977), compared with 0.911 (95%CI, 0.795 to 0.955) of LLD_predicted_ and LLD_true_.

**Fig. 3:**
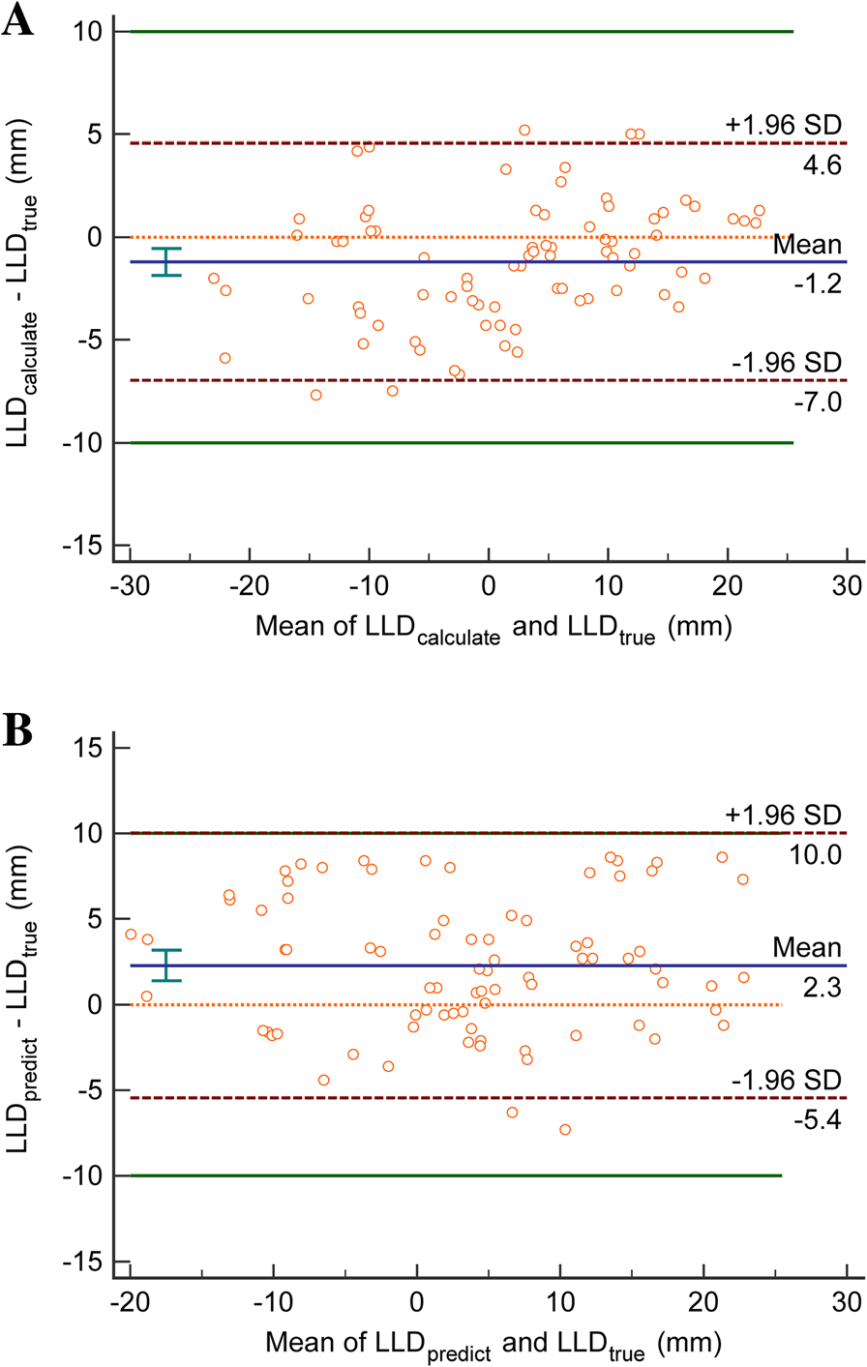
Bland-Altman plots for the disparity between the LLD_calculate_ and LLD_true_ (**a**), and between LLD_predict_ and LLD_true_ (**b**) measured by different ways. The mean bias is shown as a solid blue line; the dashed dark red lines represent the 95% limits, the green lines represent the maximum allowed clinical difference. SD = standard deviation

Bland-Altman analysis also showed good agreement between LLD_calculated_ and LLD_true_ (Fig. 3a), and between LLD_predicted_ and LLD_true_ (Fig. 3b).

LLD_predicted_ - LLD_true_ represented the total error, with an average absolute value of 3.7 (range, 0.1 to 8.6) mm. An LLD less than 10 mm will not cause obvious dysfunction and is not clinically significant [14, 16].

### RLLD values before and after surgery

The absolute values of RLLD are shown in Table 1. The average absolute values of RLLD before and after surgery were 17.4 mm and 6.3 mm, respectively, and a paired *t*-test (two-sided) showed statistically significant difference (t = 9.045, *p* < 0.001).

**Table 1:** Absolute values of RLLD

	Minimum	Maximum	Mean	Standard deviation	
Preoperative RLLD(mm)	0.6	48.6	17.4	10.3	*t* = 9.045
Postoperative RLLD(mm)	0.0	18.4	6.3	3.7	*p* < 0.001

## Discussion

This study reviewed the data of 80 cases in our hospital between May 2016 and October 2018. After measurement and calculation, the error of the calculation method itself was − 1.2 mm, which may be due to the fact that the calculation method takes into consideration the parameters of the coronal plane rather than the sagittal plane. Overall, the calculation method estimated the average error of LLD within 5 mm, which is a reliable algorithm. Furthermore, the LLD before and after surgery in DDH patients (the largest scale to date) was also investigated. Among the patients included in this study, the difference in RLLD before and after surgery was significant.

True LLD refers to the presence of bilateral lower extremity bones of unequal lengths, which is usually caused by abnormal development of the affected limb due to various pathological factors. The common associated diseases include DDH, trauma, Perthes disease, and suppurative hip arthritis. To evaluate true LLD clinically, the difference in the distance between the anterior superior iliac spine (ASIS) and the medial malleolus can be measured [26], and in imaging studies, some people measure the length difference of the legs, which is the difference in the distance from the midpoint of the distal tibia to the femoral head or the greater trochanter [10], while others measure the inclination of the pelvis [27, 28]. Variations in the length of the lower limbs may not only cause LLD but also change the angle of the pelvis. In 1983, Friberg et al. [10] demonstrated that LLD can lead to changes in the coronal and cross-sectional angles of the pelvis and spine.

However, the number of LLD measurement studies on DDH is very small. Zhang Y et al. [29] found the sacral base line was a good choice for accurate LLD measurement. After extensive research, Zhang Z et al. [24] found that the distance from the lower edge of the sacroiliac joint to the ankle, which they named the relative limb length (RLL), was a good choice for the measurement of True LLD in DDH. They also found that the length of the affected limb could be shorter or longer, but it was more likely to be longer. As the Wolff principle says bones in a healthy person or animal will adapt to the loads under which they are placed. In patients with DDH, due to abnormal development of the hip, the abnormal force of the affected limb may cause abnormal growth of the limbs, resulting in variations in limb length.

**Fig. 4:**
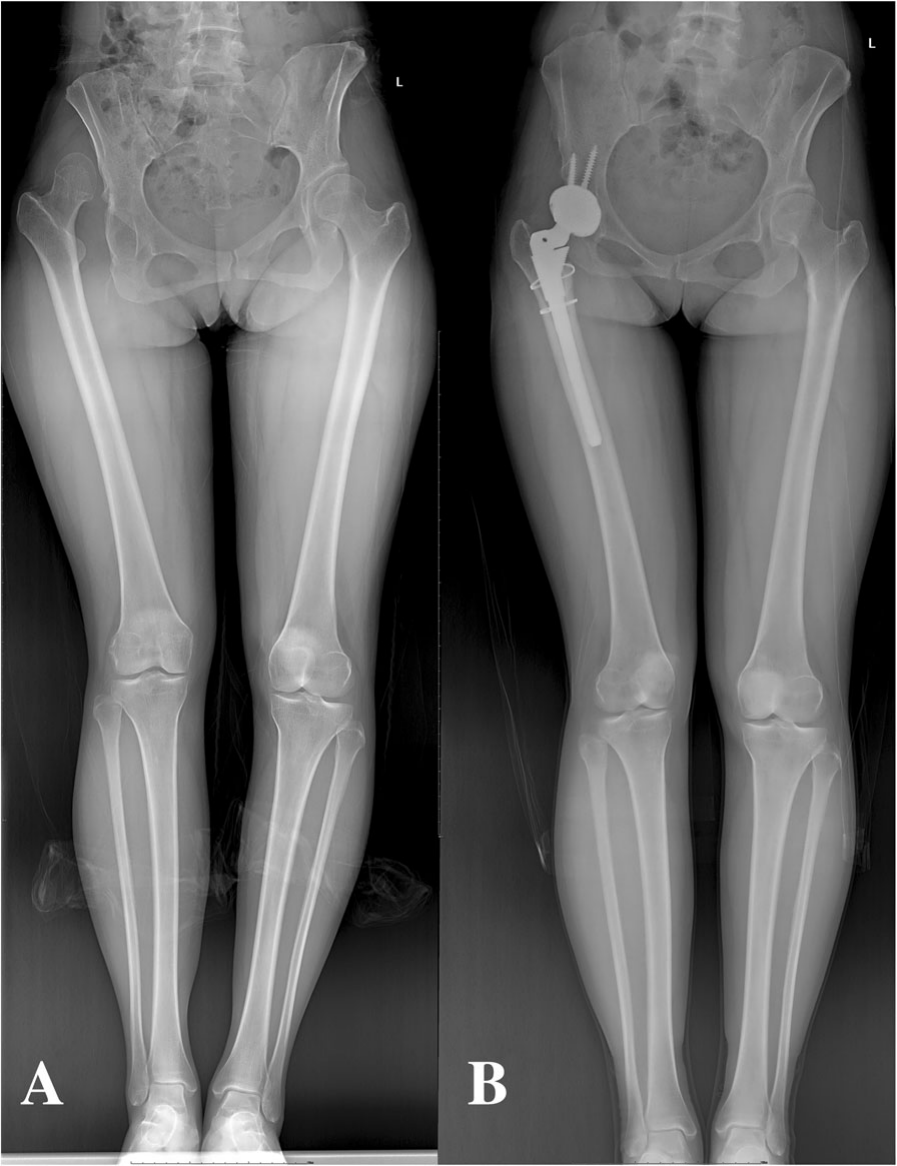
Full-length radiograph of lower limb of a Crowe-IV DDH patient. **a** The right limb is longer than the left limb preoperative. **b** The length of the affected limb is underestimated during the operation, and the length of the right limb was changed from shorter to longer postoperative

For DDH patients, in addition to the effects of LLD, various anatomical variations manifest themselves as developmental abnormalities around the hip, such as the dysplasia of the ischial ramus and the ala of ilium. These variations can also lead to muscular dysplasia and soft-tissue stiffness [30–32]. These changes will substantially increase the difficulty of THA. Accurately estimating the postoperative limb length before and during surgery is difficult. Due to the severity of preoperative deformity, some patients will require osteotomy or excessive soft tissue release to reduce the hip. Therefore, THA surgery in DDH patients is one of the most complex and challenging procedures (Fig. 4).

The calculation method proposed in this study is an effective method for accurate surgical planning and estimation of the postoperative limb length. However, this study has its limitations. Firstly, this was a retrospective study in which only patients with complete medical records were included. Secondly, the lower limb full length radiographs used here assessed the limb length in the coronal plane alone and was not 3-dimensional.

## Conclusion

In summary, the calculation method is an effective method for calculating LLD values in complex THA cases. Due to the complexity of the formula, we provide a simple tool in ‘additional file’ to help the calculation. The method promises to reduce the occurrence of severe postoperative LLD.

## Supplementary information

**Additional file 1.** The excel file which can help related calculation.

**Additional file 2.** Raw data.

## Data Availability

All data generated or analyzed during this study are included in this published article.

## Abbreviations

ASIS: Anterior superior iliac spine
COR: Center of rotation
DDH: Developmental dysplasia of the hip
ICC: Intraclass Correlation Coefficient
LL: Limb length
LLD: Limb length discrepancy
RLL: Relative limb length
RLLD: Relative limb length discrepancy
THA: Total hip arthroplasty
True LLD: True limb length discrepancy
